# General intelligence and executive functioning are overlapping but separable at genetic and molecular pathway levels: An analytical review of existing GWAS findings

**DOI:** 10.1371/journal.pone.0272368

**Published:** 2022-10-17

**Authors:** Liliana G. Ciobanu, Lazar Stankov, K. Oliver Schubert, Azmeraw T. Amare, M. Catharine Jawahar, Ellie Lawrence-Wood, Natalie T. Mills, Matthew Knight, Scott R. Clark, Eugene Aidman

**Affiliations:** 1 Discipline of Psychiatry, University of Adelaide, Adelaide, SA, Australia; 2 School of Psychology, The University of Sydney, Sydney, NSW, Australia; 3 Northern Adelaide Mental Health Services, Adelaide, SA, Australia; 4 National Health and Medical Research Council (NHMRC) Centre of Research Excellence in Frailty and Healthy Ageing, University of Adelaide, Adelaide, Australia; 5 Weapons and Combat Systems Division, Defence Science & Technology Group, Edinburgh, SA, Australia; 6 School of Biomedical Sciences & Pharmacy, University of Newcastle, Callaghan, NSW, Australia; 7 Land Division, Defence Science & Technology Group, Edinburgh, SA, Australia; University of Edinburgh, UNITED KINGDOM

## Abstract

Understanding the genomic architecture and molecular mechanisms of cognitive functioning in healthy individuals is critical for developing tailored interventions to enhance cognitive functioning, as well as for identifying targets for treating impaired cognition. There has been substantial progress in uncovering the genetic composition of the general cognitive ability (*g*). However, there is an ongoing debate whether executive functioning (EF)–another key predictor of cognitive health and performance, is separable from general *g*. To provide an analytical review on existing findings on genetic influences on the relationship between *g* and EF, we re-analysed a subset of genome-wide association studies (GWAS) from the GWAS catalogue that used measures of *g* and EF as outcomes in non-clinical populations. We identified two sets of single nucleotide polymorphisms (SNPs) associated with *g* (1,372 SNPs across 12 studies), and EF (300 SNPs across 5 studies) at p<5x10^-6^. A comparative analysis of GWAS-identified *g* and EF SNPs in high linkage disequilibrium (LD), followed by pathway enrichment analyses suggest that *g* and EF are overlapping but separable at genetic variant and molecular pathway levels, however more evidence is required to characterize the genetic overlap/distinction between the two constructs. While not without limitations, these findings may have implications for navigating further research towards translatable genetic findings for cognitive remediation, enhancement, and augmentation.

## Introduction

The heritability of cognitive performance was recognised through twin and family studies long before the development of high-throughput genotyping and genome-wide association study (GWAS) methodology [1, 2]. At the beginning of GWAS era, it was assumed that given the estimation of heritability at 50% across the lifespan, it was only a matter of time until the key gene(s) involved in cognition were identified [3]. However, cognitive genomics has proved to be a challenging area of research due to a lack of consensus on the theoretical construct of cognitive functioning and its components, and the highly polygenic nature of cognitive functioning resulting in small effects from each implicated genetic variant. Consequently, large sample sizes are required for the discovery of genetic variants associated with cognitive phenotypes at acceptable levels of statistical significance. While the problem of adequate power can be resolved by coordinating the global efforts on collecting and analysing data from various sources, the unresolved problem of definition of cognitive functioning components and the corresponding measurement instruments continues to cause the poor replicability of results, thereby limiting clinical translatability of existing GWAS findings (for a detailed review, see [4, 5]).

The recent success in identifying genetic associations of general cognitive ability, *g* [6], a cognitive construct typically derived as the first unrotated principal component of multiple cognitive test metrics, has greatly added to appreciation of the genetic complexity of cognition under the assumption that *g* captures about 25 to 40% of the total variance when a battery of multiple cognitive tests is administered to a sample with a good range of cognitive ability [7, 8]. However, due to the general nature of *g*, translatability of these findings to specific performance tasks is limited.

On the other hand, Executive Functioning (EF)—a set of high-level mental processes that are fundamental to cognitive control of behaviour [9]—is one of the most widely used psychological constructs to assess cognitive functioning in health in psychopathology [10], and understanding its underlying genetic composition is an essential step in developing tailored treatments for impaired cognition. Deficits in EF are associated with almost all psychiatric disorders, including schizophrenia, bipolar disorder, major depressive disorder (MDD), obsessive-compulsive disorder (OCD), posttraumatic stress disorder (PTSD), attention-deficit/hyperactivity disorder (ADHD) and substance use disorders [11, 12], suggesting that EF deficits could be a risk factor for, or a phenotypical feature of, general psychopathology [13, 14].

Defining and measuring EF is a challenging task that has been a topic of debate in many subdisciplines of psychological science. While there are several reasons for measurement difficulty, including poorly established construct validity and low internal and/or test–retest reliability of complex executive tasks, the major issue is the task impurity problem [15]. Systematic variance and measurement error due to task specific factors is substantial and the extraction of common variance across multiple exemplar tasks is required using multivariate statistical techniques such as confirmatory factor analysis and structural equation modeling [16]. Using a twin study design [17], it has been shown that even though the individual tasks have only moderate genetic influences (.25–.55), at the level of latent variables, where measurement error is minimised, the heritability estimates were considerably higher (over .75) [18].

Given the importance of EF in overall cognitive functioning, it is reasonable to assume that there is a substantial overlap between general *g* and EF constructs [19]. In an ongoing debate on separability of EF from *g*, some authors have found no correlation between g and EF [20–22], while others showed that EF is distinguishable from *g* at the phenotypic level and predicts behaviour above and beyond *g* [18, 23–26]. The estimation of genetic correlation between *g* and EF using classical twin study design suggests that EF is moderately to highly correlated with g (rG = .5-.9) [18, 27, 28]. Due to assumptions of the twin models, it can be useful to also estimate genetic correlations in large genome-wide samples. The first study (and only to date) estimating genetic correlations between g and EF using single nucleotide polymorphism (SNP) effects from large genome-wide associations studies (GWAS) was presented in Hatoum et al. [29]. The authors conducted a GWAS on EF using over 427,000 individuals from the UK Biobank and estimated genetic correlation with *g* using LD-regression modeling and identified 129 genome-wide significant lead variants associated with EF. They concluded that the two constructs are overlapping but genetically separable at the aggregate level with correlation estimates ranging r = .7-.8, which was comparable to what twin studies were suggesting earlier. While these findings have important implications for further research, given the aggregate level of this estimation, is it not clear what specific genetic variants are common and for *g* and EF, or whether EF and *g* can be distinguished at the individual gene or molecular pathway level. This is especially evident considering inconsistent GWAS hits that were found associated with *g* or EF in previous studies.

The aim of the current study is to extend the analysis of the relationship and characterize the molecular overlap and distinction between *g* and EF, by considering genetic markers found to be associated with either intelligence or executive functions, or both, at individual SNP level across multiple studies. To provide a comprehensive overview on the biological underpinning of phenotypic relationships between general intelligence and executive function, we analytically reviewed a subset of genome-wide association studies (GWAS) that used measures of *g* and EF as outcomes. We first identified all genetic variants that were found associated with either g or EF. Then, performed functional characterization and pathway enrichment analyses of the candidate SNPs and those in high LD for both *g* and EF to examine whether biological pathways associated with *g* and EF variants converge/diverge. Finally, we compared structural (genetic variants) and functional (biological pathways) results for *g* and EF. Our study contributes to the ongoing debate on whether executive function constitutes an aspect of general intelligence or could be considered as independent cognitive abilities and adds on a better understanding of differences and similarities of *g* and EF genetic architectures at the individual SNP level.

## Materials and methods

### Study selection process

To identify GWAS studies of interest, we used the most comprehensive NHGRI-EBI GWAS catalog that provides a publicly available curated resource of all published human GWAS findings [30]. To select GWAS studies on EF and *g*, we (1) identified all cognition-related studies using GWAS catalog search engine (data release on 2020-03-08); (2) selected studies that measured EF as an outcome using the definition proposed by Snyder, Miyake [11], we chose studies that used measures primarily working memory (Counting Span test), shifting/flexibility and inhibition/information processing assessed with Trail Making test and Wisconsin Card Sorting tests; (3) selected studies that measured *g* as an outcome, we chose studies claiming to have calculated Spearman’s *g* based on tests of verbal-numerical reasoning, a collection of various cognitive tests, Wechsler’s IQ test, and Scholastic Assessment Test. Selection of both EF and *g* studies was verified by three authors who are experts in cognitive psychology (L.S., E.A., M.K.). In order to capture all relevant *g* and EF data, no published studies were excluded on the basis of participant demographics such as age or sex.

### Selection of *g* and EF genetics variants

To identify genetic variants of interest, we selected SNPs associated with EF or *g* (p < = 5 × 10^−6^). To ensure that common variants are independent, we removed SNPs with LD and Minor Allele Frequency (MAF) at the commonly accepted threshold (r^2^> = 0.6, MAF<0.05) in *g* and EF SNP lists using LDlink tool 5.1 Release [31] (index SNPs). Then, we calculated an LD-based overlap between g and EF independent variants using LD threshold at r^2^> = 0.6, and identified SNPs that are in high LD (r^2^ > = 0.8) for both g or EF (proxy SNPs, including index SNPs) using SNiPA v3.4 (released 20 November 2020, Genome assembly: GRCh37; Genome annotation: Ensembl 87; Variant set: 1000 genomes phase 3 v 5; population: European). Although we began by searching all GWAS studies, we found no GWAS hits in no-European populations, hence the use of SNiPA genome annotation for European population is justified.

### Functional annotation and pathway enrichment analyses

To provide in-silico functional annotation of genetic correlates of sentinel and proxy EF and *g* SNPs we used SNPnexus annotation tool at https://www.snp-nexus.org/v4/, Genome assembly: GRCh37. We then conducted pathway enrichment analyses separately for EF and *g* SNPs (for GWAS sentinel SNPs and those in high LD (r2> = 0.8) using the hypergeometric over-representation pathway analyses were conducted in SNPnexus tool; SNPnexus uses the Reactome knowledgebase to link the gene variants specific to, or shared by the cognitive domains in this analysis with their biological pathways [32, 33]. Statistical significance of the pathway was calculated using the Fisher’s Exact Test for all the genes involved in the original query set [34–38]. To examine possible pleiotropic effects of *g* and EF genetic variants, we scanned the GWAS catalog for any association of the SNPs of interest with other physical, psychological, and neuropsychiatric traits at p < 5 × 10^−8^.

### Terms frequently used in Genome-Wide Association Studies (GWAS)

#### Linkage Disequilibrium (LD)

Non-random association of alleles of different loci in a given population. Loci are considered to be in LD when the frequency of association of their different alleles is higher or lower than what would be expected if the loci were independent and associated randomly [39].

#### Minor Allele Frequency (MAF)

Frequency at which the second most common allele occurs in a given population. MAF is widely used in GWAS because it provides information to differentiate between common (MAF>0.05) and rare (MAF<0.05) variants in the population [40].

#### Pathway enrichment analyses

Statistical technique that help to gain mechanistic insight into gene/SNP lists generated from genome-scale experiments. This method identifies biological pathways that are enriched in a gene list more than would be expected by chance.

For further reading on GWAS methodology, please refer to the [41].

## Results

### Study selection

Our initial search for the term ‘cognition’ (EFO_0003925, mapped MeSH:D003071, 14 cognition-related traits) and an additional 11 cognition-related terms that were relevant for cognitive functioning (S1 Table) identified 54 unique studies in total (S2 Table). After a rigorous study selection process led by cognitive psychology and psychiatry experts (L.S., E.A., M.K., S.C.), we identified 17 studies that were conducted on healthy individuals and measured general cognitive ability, *g* (n = 12), or executive functioning (EF) as an outcome (n = 5). The reason for excluding studies on clinical populations is our attempt to provide a picture of relatively healthy cognition, as genetic variants associated with cognitive functioning in clinical groups may represent illness-specific associations. However, it is worth noting that some studies included in this work utilized a population study design, without specific screening for psychopathology, therefore, we cannot rule out a possibility of some pathology in our sample. This sample is characterised by a population level demographics, i.e., both sexes with age range from as early as 6 years old in a Childhood Intelligence Consortium, CHIC [42] to 102-year-old participants in CHARGE and COGENT consortia, and UK Biobank [43]. More characteristics of these studies are presented in Table 1 (more details in S2 Table).

**Table 1:** GWAS studies included.

Study	PubMed ID	Outcome measure reported	Sample size	Ancestry
**General intelligence, g, studies**				
Kornilov, Tan [44]	31620175	General cognitive ability	354	Saudi Arabian
Coleman, Bryois [45]	29520040	HiQ vs Spearman’s *g*	87,740	European
Davies, Lam [43]	29844566	General cognitive function, *g*	300,486	European
Savage, Jansen [46]	29942086	General cognitive function, *g*	269,867	European
Zabaneh, Krapohl [47]	29731509	General cognitive function, *g*	1,238	Caucasian
Sniekers, Stringer [48]	28530673	Spearman’s *g*	78,308	European
Trampush, Yang [49]	28093568	General cognitive function, *g*	35,298	European
Davies, Marioni [50]	27046643	General cognitive function, *g*	112,151	European
Davies, Armstrong [51]	25644384	General cognitive function, *g*	53,949	European
Kirkpatrick, McGue [52]	25383866	General cognitive function, *g*	3,264	European
Benyamin, Pourcain [42]	23358156	General cognitive function, *g*	17,989	European
Luciano, Hansell [53]	21130836	Information processing, *g*	4,038	European
**Executive functioning, EF, studies**				
Hatoum [29]	NA	Executive function	427000	European
Donati, Dumontheil [54]	31598132	Latent EF measures of WM and IC	4611	European
Zhang, Zhou [55]	30134085	Cognitive flexibility	4873	AA and EA
Ibrahim-Verbaas, Bressler [56]	25869804	Executive function, PS	21,860	European
Ising, Mather [57]	24629169	Information processing	890	European

Abbreviations: HiQ–high intelligence, WM–working memory, IC–inhibitory control, PS–processing speed

### Overlap between index SNPs for g and EF

**Fig 1:**
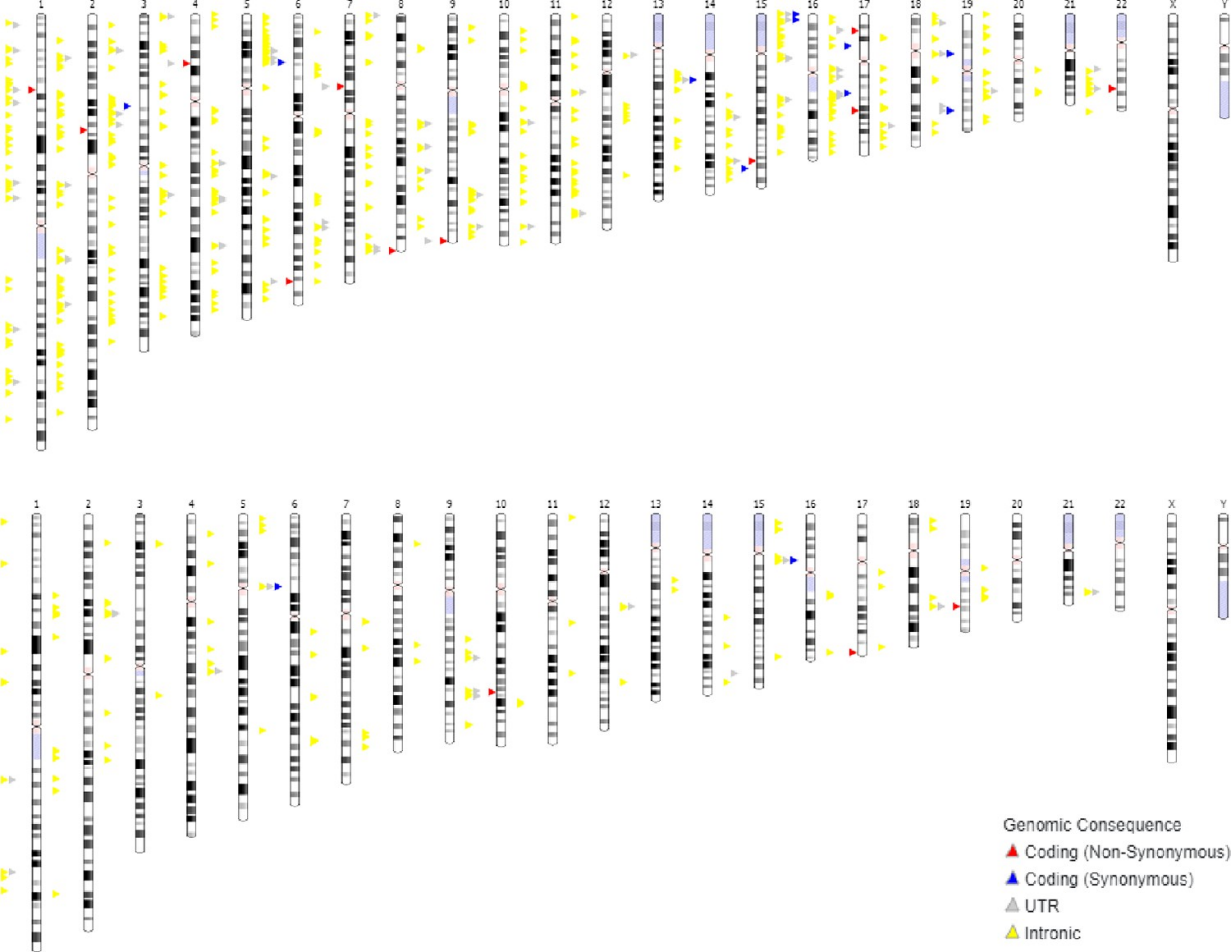
Karyotypes of genomic coordinates for *g* (A) and EF (B) index SNPs. NOTE: Sentinel SNPs were defined at LD threshold r^2^> = 0.6.

Across 17 studies included in these analyses, we identified 1,993 genetic variants that were previously found to be associated with general intelligence (general cognitive ability), *g*, across 12 studies (S3 Table), and 351 genetic variants associated with executive function, EF at the genome-wide association threshold (p<5x10^-6^) across 5 studies (S4 Table). Given that these index SNPs were obtained from different GWA studies, they may not be independent at accepted LD threshold of r^2^< = 0.6. Therefore, we performed LD pruning at r^2^< = 0.6 and MAF> = 0.5 for both g and EF lists of SNPs. This resulted in 1,372 variants for *g* and 300 variants for EF (S3 and S4 Tables). The karyotypes of genomic coordinates for *g* and EF -associated variants are presented in Fig 1.

To estimate LD-based genetic overlap between *g* and EF, we used a threshold of r^2^> = 0.6. Out of a total of *g* and EF-associated 1,672 common variants, 76 SNPs were within this threshold, suggesting a 4.6% overlap between *g* and EF. The estimated overlap was not uniform across chromosomes. Thus, there was no overlap observed for chromosomes 9, 11, 12, 14, 15, 21. For other chromosomes the overlap ranged from 1.4% (Chr 4) to 10.6% (Chr 20). More details on the estimated per chromosome overlap between *g* and EF is available in Table 2. The 23 SNPs, which are 1.4% of the total number of SNPs (n = 1,672) were common (the same) for both *g* and EF. Characteristics of *g* and EF common variants are in Table 3.

**Table 2:** Estimated genetic overlap between g and EF per chromosome.

Chr	*g* SNPs, n	EF SNPs, n	r^2^> = 0.6, n (%)
1	106	28	6 (4.5)
2	160	33	9 (4.7)
3	117	23	10 (7.1)
4	68	6	1 (1.4)
5	103	19	5 (4.1)
6	125	24	5 (3.4)
7	92	30	11 (9)
8	43	16	3 (5.1)
9	46	4	0 (0)
10	43	29	7 (9.7)
11	50	5	0 (0)
12	54	6	0 (0)
13	38	5	1 (2.3)
14	29	5	0 (0)
15	30	4	0 (0)
16	65	21	5 (5.8)
17	79	10	4 (4.5)
18	32	5	1 (2.7)
19	20	11	1 (2.2)
20	37	10	5 (10.6)
21	8	1	0 (0)
22	27	5	2 (6.3)

Note: Number of SNPs per chromosome identified at p<5x10^-6^; overlap estimated as number of g and EF SNPs in LD at r^2^> = 0.6 per chromosome; percentage calculated out of total number of SNPs for g and EF per chromosome.

**Table 3:** Structural characteristics and predicted functional consequences of 23 index SNPs common for general intelligence (*g*) and executive function (EF).

SNP	Chr	Position	MAF	Risk Allele	Predicted function	OG
rs13019832	2	60710571	A = 0.279	A	intronic	*BCL11A*
rs17654195	2	137409714	A = 0.121	na	none	none
rs4500960	2	162818621	T = 0.493	T	intronic	*SLC4A10*
rs6741949	2	162910223	C = 0.265	C	intronic	*DPP4*
rs9851068	3	23839884	G = 0.426	A	none	none
rs17518584	3	85604923	C = 0.482	T	intronic	*CADM2*
rs6881733	5	92586991	T = 0.392	T	none	none
rs11759522	6	3450814	G = 0.455	C	intronic	*SLC22A23*
rs4716325	6	19025741	C = 0.341	C	none	none
rs13197257	6	128333682	T = 0.176	T	intronic	*PTPRK*
rs55658584	7	104994721	A = 0.112	A	intronic	*SRPK2*
rs6467482	7	132918345	A = 0.347	A	none	none
rs35284403	7	132948884	C = 0.292	T	intronic	*EXOC4*
rs6982152	8	64779013	T = 0.143	na	intronic	*AC022639*.*1*
rs13262595	8	143316970	A = 0.24	A	intronic	*TSNARE1*
rs4148398	10	101592622	A = 0.263	na	intronic	*ABCC2*
rs11596211	10	101840119	A = 0.171	A	intronic	*CPN1*
rs749694	10	103519784	G = 0.446	A	none	none
rs2735421	10	103541016	T = 0.403	T	none	none
rs61874768	10	103880118	T = 0.071	T	5 prime UTR	*LDB1*
rs35937770	17	44808360	A = 0.2	na	intronic	*NSF*
rs17698176	17	44819595	G = 0.082	T	intronic	*NSF*
rs10119	19	45406673	A = 0.248	na	3 prime UTR	*TOMM40*

Abbreviations: Chr–chromosome, MAF–Minor Allele Frequency, OG—Overlapped Gene, SNP–Single Nucleotide Polymorphism

Pathway enrichment analyses using Reactome knowledgebase identified nine molecular pathways associated with *g* index variants at FDR<0.05. These pathways are known to be involved in neuronal system synaptic activity (transmission across chemical synapses, protein-protein interaction at synapses, neurotransmitter receptors and postsynaptic signal transmission, synaptic adhesion-like molecules, activation of NMDA receptors and postsynaptic events), developmental biology (EPHA-mediated growth cone collapse), immune system (Butyrophilin (BTN) family interactions), and cell-to-cell communication (Adherens junctions interactions) (S3 Table). No statistically significant pathways for EF index variants were identified under p<0.05.

**Fig 2:**
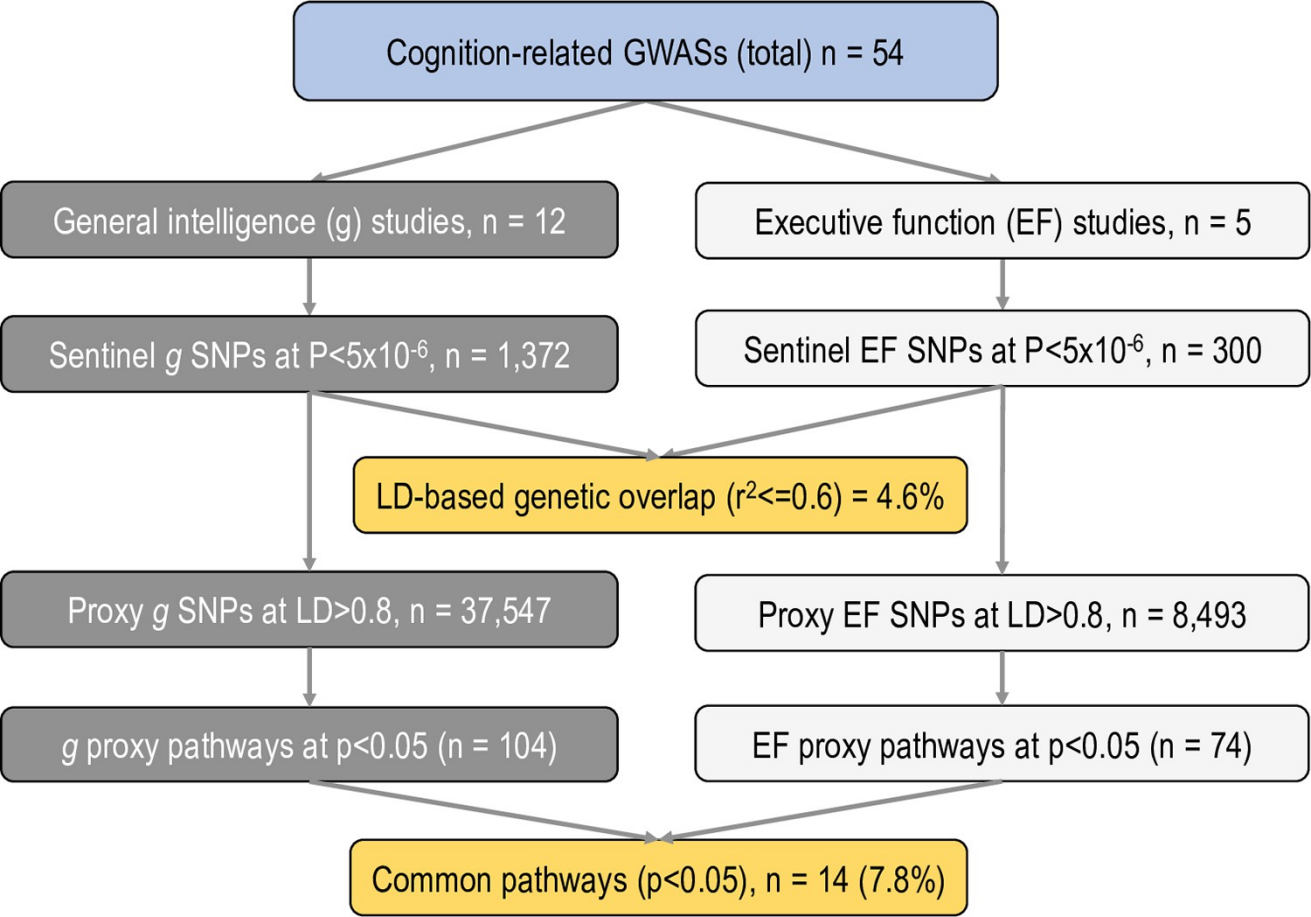
Summary of the findings. NOTE: Sentinel SNPs were defined as independent at LD threshold r^2^> = 0.6 for g and EF. The overlap between f and EF independent sentinel variants was determined as a proportion of SNPs at LD threshold r^2^> = 0.6 between the *g* and EF-associated SNPs.

Fig 2 provides summarises the findings of the current study.

Scanning of the GWAS catalog database for associations of the 23 *g* and EF common SNPs with other traits revealed that 8 SNPs have been previously associated with biological (hippocampal [58] and other brain regions volume [59], cortical surface area [60], mean reticulocyte volume [61], serum type 1 collagen metabolite levels [62], cerebral amyloid deposition [63]), psychological, psychiatric and neurological traits (anxiety & neuroticism [64], schizophrenia [65, 66], family history of Alzheimer’s disease [67]) cognition-related traits; as well as being associated with other behavioural and functional traits (asthma [68], alcohol consumption [69], sedentary lifestyle [70], household income [71]) (Table 4).

**Table 4:** Pleiotropic associations of common *g* and EF SNPs.

SNP	Risk allele	P-value	Mapped gene	Reported trait	Study accession
rs13019832	A	1 x 10^−8^	*BCL11A*	Household income	GCST009524
rs6741949	G	2 x 10^−9^	*DPP4*	Asthma	GCST010043
	G	3 x 10^−7^	*DPP4*	Hippocampal volume	GCST001485
rs6982152	C	4 x 10^−15^	*LINC01414*	Alcohol consumption (drinks per week)	GCST007472
rs13262595	na	3 x 10^−14^	*TSNARE1*	Schizophrenia	GCST009337
	A	3 x 10^−13^	*TSNARE1*	Anxiety	GCST006478
	A	6 x 10^−9^	*TSNARE1*	Neuroticism	GCST006476
	na	1 x 10^−12^	*TSNARE1*	Leisure sedentary behaviour (computer use)	GCST010085
	na	5 x 10^−9^	*TSNARE1*	Schizophrenia	GCST010645
rs61874768	T	4 x 10^−18^	*LDB1*	Mean reticulocyte volume	GCST90002396
rs35937770	A	2 x 10^−11^	*NSF*	Cortical surface area	GCST010282
rs17698176	T	2 x 10^−8^	*NSF*	Neuroticism	GCST006476
	na	5 x 10^−9^	*NSF*	Brain region volumes	GCST009518
rs10119	na	1 x 10^−307^	*TOMM40*	Family history of Alzheimer’s disease	GCST005921
	A	3 x 10^−8^	*TOMM40*	Serum type 1 collagen metabolite levels	GCST011622
	A	4 x 10^−10^	*TOMM40*	Cerebral amyloid deposition (PET imaging)	GCST006904

NOTE: In this table, only variants that are common for both g and EF and have reported GWAS pleiotropic associations are shown.

### *g* and EF molecular pathways

To examine what biological pathways are overrepresented for *g* and EF variants and specify common and/or distinct *g* and EF pathways, we conducted an enrichment pathway analysis for *g* and EF SNP lists using SNPnexus annotation tool. To improve signal detectability, we ran our analyses under the commonly accepted assumption that highly correlated genetic variants are likely to be involved in the same biological processes. For 1,372 independent index *g* SNPs we identified 37,547 proxy SNPs (r^2^>0.8) (S5 Table) and for 300 EF index SNPs we found 8,493 proxy SNPs (r^2^>0.8) (S6 Table).

To eliminate reporting bias, we report on *g* and EF pathways at various nominal thresholds. Thus, under a relaxed *p*<0.05 we found 104 *g-*associated pathways and 74 EF-associated pathways with 7.8% (n = 14) being common for both constructs (Table 5). At the *p*<0.01 there were 75 and 24 *g* and EF pathways respectively with only 2 common pathways between the two (2.9%). At the most stringent threshold of p<0.002 we found 12 *g*-related and only one EF-related pathway (MECP2 regulates transcription factors) with no common pathways identified (Table 6, also see S6 Table). No pathways survived FDR correction for multiple testing. This suggests that statistical threshold plays an important role when examining and interpreting molecular overlap between two psychological constructs.

**Table 5:** Common pathways associated with *g* and EF-proxy SNPs (p<0.05).

Reactome pathway description	Parent	P-value	
		g proxy	ef proxy
Neurotransmitter receptors and postsynaptic signal transmission	NS	0.001	0.034
GPCR downstream signalling	SD	0.002	0.038
*Chromatin modifying enzymes	**ChO**	0.004	0.005
*Chromatin organization	**ChO**	0.004	0.005
Activation of NMDA receptors and postsynaptic events	NS	0.005	0.025
Synthesis of IPs in the nucleus	M	0.013	0.046
TET1,2,3 and TDG demethylate DNA	GE(T)	0.013	0.046
Circadian Clock	CC	0.019	0.049
MECP2 regulates transcription factors	GE(T)	0.021	0.001
Class I MHC mediated antigen processing & presentation	IS	0.048	0.042
Defective RFT1 causes RFT1-CDG (CDG-1n)	D	0.048	0.012
Defective ABCC2 causes Dubin-Johnson syndrome	D	0.048	0.012
Oleoyl-phe metabolism	M	0.048	0.012
RUNX1 regulates genes involved in megakaryocyte differentiation	GE(T)	0.050	0.027

NOTE: In this table, we report the common g and EF-associated pathways under p<0.05. The pathways that were also common under p<0.01 are highlighted in bold and marked with *asterisks.

Abbreviations: ChO—Chromatin organization, D–Disease, GE(T)—Gene expression (Transcription), IS—Immune System, M–Metabolism, NS—Neuronal System, SD—Signal Transduction

**Table 6:** Specific g and EF pathways associated with *g* and EF-proxy SNPs (p<0.002).

Reactome pathway description	Parent	P-value
***g pathways***	NS	
Neuronal System	IS	0.0000
Butyrophilin (BTN) family interactions	NS	0.0000
Synaptic adhesion-like molecules	NS	0.0000
Protein-protein interactions at synapses	NS	0.0001
Transmission across Chemical Synapses	ST	0.0001
Olfactory Signaling Pathway	ST	0.0002
G alpha (s) signalling events	ST	0.0004
GPCR ligand binding	C-Cc	0.0007
Adherens junctions interactions	NS	0.0008
Neurotransmitter receptors and postsynaptic signal transmission	ST	0.0013
GPCR downstream signalling	ST	0.0018
Signaling by GPCR		0.0019
***EF pathways***		
MECP2 regulates transcription factors	GE(T)	0.0013

Abbreviations: C-Cc–Cell-Cell communication, GE(T)—Gene expression (Transcription), IS—Immune System, NS—Neuronal System, SD—Signal Transduction

## Discussion

An ongoing debate on whether executive functions constitute an aspect of general intelligence, *g*, or could be considered as independent cognitive abilities is an important research question for both basic research and its clinical applications that will provide clarity about the hierarchy of cognitive constructs and the validity of their measurement.

In the current study, we have discovered new aspects of the relationship between executive functioning and general intelligence by examining the genetic markers associated with either intelligence or executive function, or both, at an individual gene level.

While some earlier studies failed to find correlation between intelligence and executive functioning, more recent findings suggest both overlapping and separable aspects of *g* and EF constructs. Here, we found that despite the ‘clouded’ phenotypes of *g* and EF typically defined using a mixture of cognitive tests, the genetic architecture of these constructs appears to be overlapping but separable. In view of the limited genetic information available to date, our results suggest that *g* and EF are separable at the GWAS-identified SNP level (4.6% LD-based overlap at r^2^< = 0.6). These results should be treated under an assumption of statistical interactions [72], which were not tested in this study: a variant may be significant for one trait but not the other, but that does not mean that its effect sizes for the two traits are significantly different. NOTE: These results should not be compared with previously reported aggregate findings on much larger genetic correlation between g and EF; in our study, we provide a comprehensive overview of individual variants that have been identified as implicated by previous GWAS studies, reflecting, therefore, a current status of our knowledge about individual SNPs associated with g or/and EF, rather than on aggregated estimates. Considering high aggregate estimates of genetic correlation between *g* and EF, we can expect to identify more individual markers in the future with more high-quality studies of sufficient power and refined phenotypes, which will inevitably enrich findings reported here. Furthermore, the contribution of rare variants not tagged by SNP arrays is a further potential source of underestimation in existing GWASs. Factoring into *g* and EF biological pathways, we observed a 7.8% overlap defined as a proportion of common pathways at nominal p<0.05. The small increase in biological pathways overlap compared to genetic overlap between *g* and EF can be partially explained by pleiotropic effects of identified genetic variants on the downstream molecular signaling and cellular functioning. This might also be partially attributed to increase of genetic information fed into pathways enrichment algorithm by proxy variants in high LD at r^2^> = 0.8, however, we argue that by limiting our analyses to sentinel GWAS SNPs, we are likely missing downstream biological effects of proxy variants that despite proximity and high correlation with sentinel variants, might have different functional consequences.

Most of the biological pathways that were common for *g* and EF (at p<0.05) were involved in basic neuronal and cellular functioning processes, such as signal transduction, gene expression (transcription), and metabolism, which one could expect given the common biological grounds for *g* and EF; however, the role for *Circadian clock* and *Class I MHC mediated antigen processing & presentation* (adaptive immune system response) pathways in *g* and EF is less obvious.

Many studies have examined the relationship between circadian clock and cognition, as well as between cognition and the immune system, however, the exact molecular mechanism linking all three: circadian clock, immune system response, and cognitive functioning has not been established yet. It has been previously shown that MHC class I immune proteins are critical for hippocampus-dependent memory formation [73] and for maintaining neuronal structural complexity [74]. On the other hand, immune functions are long known to be important regulators of circadian rhythms [75]. Taken together, our findings are consistent with previous observations that there is a relationship between the circadian clock, cognition, and the immune response [76, 77], which appears to be important for both *g* and EF.

The two common biological pathways for *g* and EF constructs, *Chromatin organization* and *Chromatin modifying enzymes*, that remained statistically significant at both relaxed p<0.05 and more stringent p<0.01, are of particular interest. For decades, research has implicated epigenetic mechanisms, such as histone modifications in regulating chromatin compaction necessary for experience-dependent changes to gene expression and cell function during memory formation [78, 79], however little is known the role epigenetic mechanisms play in general intelligence or/and executive functioning. Our study suggests that chromatin organization molecular processes, which regulate the accessibility of DNA and help to protect it from damage, are related to both g and EF via chromatin modifying enzymes pathway as a regulatory mechanism underlying long-lasting changes in neurons, with direct implications on brain function [80].

Apart from common g and EF pathways we also looked to identify those specific for *g* or EF, to suggest molecular mechanisms that distinguish the two constructs. We found that that mitochondria-specific autophagy (*mitophagy*), a fundamental process that contributes to mitochondrial quality control by selectively eliminating dysfunctional mitochondria [81], was associated with EF but not *g*. Previous research supports the role of mitochondrial functioning in cognition without suggesting specific domains [82–84]. The *plasma lipoprotein* pathway, integral to energy and cholesterol metabolism in cells, including neurons, and NR1H2 and NR1H3-mediated signaling, that regulates gene expression linked to cholesterol transport and efflux, appear to be EF-specific. Although this is consistent with previous clinical observations suggesting a link between executing functioning and cholesterol [85, 86], more work required to understand this relationship. Although *cell cycle* pathways were prominent in *g* and not EF, given their central role in the neuronal life cycle [87], and the greater power of *g* GWAS included in this analysis, potentially larger EF-based studies may find similar relationships. Studies in clinical populations have found associations between cell cycle genes and EF [88] (for more details please see S4 and S6 Tables). The top EF-associated pathway *MECP2 regulates transcription factors* (p = 0.0013) and is known to be a critical regulator of chromatin in neurodevelopment and adult brain function [89]. *MECP2* was also associated with *g* at the p = 0.02.

Consistent with patterns typical for complex traits [90], we observed pleiotropic effects of *g* and EF shared genetic variants with other physical, psychological and neuropsychiatric traits. While association with some traits, like cortical surface area, hippocampal and other brain region volumes, schizophrenia, and others is explainable by a substantial cognitive load in these traits, the associations with asthma, sedentary lifestyle (computer use), household income, and others are less intuitive. However, a new line of research on the effects of the genetic contribution to cognitive functioning is emerging. Thus, a recent study estimated the association of types of screen time (watching TV and online videos, socializing via social media, text, and video chat, and gaming) with intelligence after accounting for screen type, socioeconomic status, and genetic predisposition for intelligence. Surprisingly, gaming and watching was shown positively influence the amount of change in intelligence so that children who played more video games at 9–10 years of age showed the more gains in intelligence two years later [91]. Further research addressing these pleiotropic effects would be required to unpack the mechanisms of these relationships.

### Limitations and future directions

This study has several limitations. First, it shares the common limitations of the included GWAS studies. While we attempted to ‘clean’ the *g* and EF phenotypes by selecting studies that used comparable cognitive measures, the opportunistic nature of phenotyping in different studies influences the replicability of GWAS results and, therefore, our conclusions. Second, while there is agreement on how to define *g*, the definition of EF is more debatable. Previous studies have used single tasks to measure EF, however, this approach leads to the inclusion of task specific variance, when factor analysis across multiple tasks may more accurately distil a core measure. Task specific variance may explain the difference between the derived sumstats from single task studies and the approach taken by Hatoum et al. [29] who used confirmatory factor analysis for their EF GWAS. Third, our estimate of genetic overlap 76/1672 = 4.6% is based on GWAS significant SNPs and should not be considered as an estimate of genetic correlation, which is computed by using whole genome genetic variants. While we observed that *g* and EF appear to be overlapping but largely separable at an individual SNP level, the estimation of aggregated genetic effects on *g* that are correlated with genetic effects on EF independently of the heritability estimates of the two traits, derived from the genetic analysis of covariance (such as LD Score Regression) [29], shows a much larger genetic correlation between g and EF (r = 0.7–0.8). Power limitations in current GWASs dictate that SNPs reaching significance represent only a fraction of those that impact on complex phenotypes, hence we are able to explain less of the variance. This discrepancy can also be attributed to the differences in methodology used; specifically, given the review nature of this work, the *g* and EF studies here were selected based on different representations of the constructs, while [29] used a single measure of *g* and EF. We suggest that the inconsistency in EF-related pathways we observed is complementary to the aforementioned findings, with the two distinct methodologies producing different perspectives. EF is a highly complex phenotype, and it is unlikely that only several pathways are involved in its underlying processes. We suggest that with increased power in GWAS and improved methodology we will be able to identify even more pathways involved in EF.

Despite the numerous theoretical models, large inconsistencies in defining *g* and EF in GWAS studies, where the same measures used for both constructs [92], remain a major challenge for the statistical estimation of genetic overlap between *g* and EF. Furthermore, given the limited statistical power for the majority of EF studies to date, our results should be treated with caution as with better-powered EF studies the relationship between g and EF might change.

To overcome these limitations, it is important to examine genetic and functional relationships between the smallest units of measurement characterising cognitive functioning–individual cognitive test metrics. Such examination should enable a biology-informed measurement model of cognitive functioning. We view this approach as a comprehensive mapping of broad and specific cognitive functions to their genetic correlates, which might enable uncovering of a hierarchical structure of cognition and suggest novel targets for functional interventions with translation potential.

### Conclusions

The question of separability of executive function from general intelligence has been an ongoing debate for decades. In this study, we examine biological underpinnings of these cognitive constructs and analyse high level insights into genetic architectures of *g* and EF and their functional consequences by employing recent GWAS findings and pathways enrichment analyses. We found that while some genetic variants are common for *g* and EF, executive functions appear to be separable from general intelligence at both structural and functional levels; however more evidence is required to characterize the genetic overlap/distinction between *g* and EF. To the best of our knowledge, this study is the first to systematically compare structural and functional genetic correlates of general intelligence and executive function at an individual SNP level across multiple studies. It provides biologically tractable evidence to inform cognitive enhancement programs focused on modifiable executive functions and can serve as a guide for future research in the field.

## Supporting information

S1 Table GWAS catalog cognition related terms. (DOCX)

S2 Table Cognition related GWAS studies. (XLSX)

S3 Table Index g SNPs and pathways. (XLSX)

S4 Table Index EF SNPs and pathways. (XLSX)

S5 Table Proxy g SNPs and pathways. (XLSX)

S6 Table Proxy EF SNPs and pathways. (XLSX)
