# Supplementary material for: General intelligence and executive functioning are overlapping but separable at genetic and molecular pathway levels: An analytical review of existing GWAS findings
Source: PLoS One. 2022 Oct 17;17(10):e0272368. doi: 10.1371/journal.pone.0272368 (PMC9576059; doi:10.1371/journal.pone.0272368)
Supplement: S1 Table — (DOCX) [file pone.0272368.s001.docx]

Supplementary Material

**General intelligence and executive functioning are separable at genetic and molecular pathways levels: an analytical review of previous GWAS findings**

Liliana G Ciobanu^1^, Lazar Stankov^6^, K Oliver Schubert^1,2^, Azmeraw T Amare^1,3^, M Catharine Jawahar^1^, Ellie Lawrence-Wood^1^, Natalie T Mills^1^, Matthew Knight^1,5^, Scott R Clark^1^, Eugene Aidman^4,6,7^

^1^Discipline of Psychiatry, University of Adelaide, SA

^2^Northern Adelaide Mental Health Services, SA

^3^South Australian Health and Medical Research Institute (SAHMRI), SA

^4^School of Biomedical Sciences & Pharmacy, University of Newcastle, NSW

^5^Weapons and Combat Systems Division, Defence Science & Technology Group, Edinburgh, SA
^6^School of Psychology, The University of Sydney, NSW

^7^Land Division, Defence Science & Technology Group, Edinburgh, SA

Corresponding author: [liliana.ciobanu@adelaide.edu.au](mailto:liliana.ciobanu@adelaide.edu.au)

**Table S1.** Cognition-related terms in GWAS catalog

| **Cognition traits** | **Description** | **Studies** | **Associations** |
| --- | --- | --- | --- |
| *Reported traits* |  |  |  |
| Cognitive function  Residual cognition | Intellectual or mental process whereby an organism becomes aware of or obtains knowledge | 76 | 6029 |
| *Child traits* |  |  |  |
| Infant expressive language ability | Mental process necessary for language acquisition in infancy | 1 | 8 |
| Information processing speed | Time taken to analyse or transform input information in order to produce information as output | 12 | 55 |
| Intelligence | The ability to learn and to deal with new situations and to deal effectively with tasks involving abstractions | 27 | 2967 |
| Mathematical ability | Mental process necessary to acquire knowledge or skill in the measurement, properties, and relationships of quantities and sets, using numbers and symbols (mathematics) | 10 | 2743 |
| Musical aptitude |  | 1 | 0 |
| Non-word reading | Complex cognitive process of decoding symbols in order to derive that they do not form meaningful words | 2 | 28 |
| Perception of facial expression | NA | NA | NA |
| Reading | Complex cognitive process of decoding symbols in order to construct or derive meaning | 3 | 30 |
| Reading and spelling ability | Complex cognitive processes involved in assembling and decoding symbols in order to construct or derive words | 13 | 114 |
| Reasoning | Thinking that is coherent and logical, using knowledge to evaluate the truth value of a proposition | 3 | 16 |
| Receptive language perception | The ability to understand or comprehend language heard or read | 1 | 0 |
| Speech perception | The process whereby an utterance is decoded into a representation in terms of linguistic units (sequences of phonetic segments which combine to form lexical and grammatical morphemes) | 1 | 2 |
| Word reading | Complex cognitive process of decoding symbols in order to construct or derive meaningful words | 2 | 32 |
| *Additional traits* |  |  |  |
| Memory performance | Quantification of a subject's activities involved in the mental information processing system that receives (registers), modifies, stores, and retrieves informational stimuli | 28 | 181 |
| Verbal memory measurement | Quantification of an individual's ability to store and recall linguistic information | 1 | 3 |
| Word list delayed recall measurement | Word list delayed recall is a verbal declarative memory test used to quantify memory performance | 1 | 35 |
| Executive function measurement | Quantification of executive function in an individual, for example using the Behavioural Rating Inventory of Executive Function (BRIEF) | 7 | 26 |
| Verbal-numerical reasoning measurement | Quantification of verbal and/or numerical reasoning, typically based on a written or multiple choice test | 1 | 0 |
| Attention function measurement | Quantification of an individual's attention function through administration of one or more standardised tests such as the Connors Continuous Performance Test-II (CPT-II) | 4 | 42 |
| Intra-individual reaction time variability measurement | Quantification of the variability in reaction time (the interval of time between the presentation of a stimulus to a subject and the beginning of the subject's response to that stimulus) of an individual, over successive tasks | 2 | 2 |
| Language measurement | Quantification of some aspect of language or language development | 8 | 21 |
| Psychomotor performance | The coordination of a sensory or ideational (cognitive) process and a motor activity | 1 | 1 |
| Reaction time measurement | Quantification of the interval of time between the presentation of a stimulus to a subject and the beginning of the subject's response to that stimulus | 3 | 524 |
| Cognitive function measurement | Quantification of some aspect of cognitive function | 20 | 902 |

NOTE: This list of cognition-related terms represents our first unbiased search of all conducted GWASs on any cognition-related trait. This was necessary in attempt to overcome one of the major limitations of inconsistent labelling of cognitive processes and underlying measurement instruments.
